# Cartilage-hair hypoplasia in a patient with compound heterozygous variants in the *RMRP* gene: A case report

**DOI:** 10.1097/MD.0000000000047005

**Published:** 2026-01-09

**Authors:** Shuangzhu Lin, Qiandui Chen, Yangfan Qi, Xiaoyu Sun, Wanqi Wang, Kai Jiang, Xinyu Zhou

**Affiliations:** a College of Traditional Chinese Medicine, Changchun University of Chinese Medicine, Changchun, Jilin Province, China; b Pediatric Department, Gansu Provincial Hospital of Traditional Chinese Medicine, Lanzhou, Gansu Province, China; c College of Traditional Chinese Medicine, Changchun University of Chinese Medicine, Changchun, Jilin Province, China; d College of Traditional Chinese Medicine, Changchun University of Chinese Medicine, Changchun, Jilin Province, China.

**Keywords:** cartilage-hair hypoplasia, child, gene, RMRP, short stature

## Abstract

**Rationale:**

Cartilage-hair hypoplasia (CHH) is an autosomal recessive disorder caused by homozygous or compound heterozygous mutations in the *RMRP* gene, which is extremely rare in the population, and its most common feature is disproportionately short limb shortness with short and thickened long bones, usually found in newborns, occasionally found in the prenatal stage, and other clinical features include a series of extracutaneous manifestations, such as hypoglycemia, gastrointestinal dysfunction, immunodeficiency, anemia and increased risk of malignant tumors, etc, the specific pathogenesis is unknown.

**Patient concerns:**

The case of a 1.5-year-old male patient with severe short stature (height 70 cm, <−3 SD), sparse scalp hair, and short limbs, without ligamentous laxity or anemia.

**Diagnoses:**

Family-based whole-exome sequencing revealed compound heterozygous variants in *RMRP*: NR_003051.3: n.-21_-9dup and n.5C > T. Classified as pathogenic or likely pathogenic according to ACMG guidelines, these variants confirm the diagnosis of CHH.

**Interventions:**

Due to the elevated tumor predisposition associated with CHH, and because growth hormone therapy is currently contraindicated, no disease-specific interventions have been initiated.

**Outcomes:**

We have confirmed that the child has been diagnosed with CHH, but no intervention has been given, and we will do further follow-up and look forward to finding a treatment for the disease.

**Lessons:**

This report describes a rare case of *RMRP*-associated CHH. This case broadens the clinical understanding of the *RMRP* mutational spectrum and phenotypic variability, providing new insights into genotype-phenotype correlations in RMRP-associated disorders. We were currently unable to take effective treatment measures for this child, and we hope that in the future, there will be treatments such as gene therapy to bring hope to children and their families.

## 1. Introduction

Cartilage-hair hypoplasia (CHH) is an autosomal recessive disorder caused by homozygous or compound heterozygous mutations in the RNA Component of the mitochondrial RNA processing endoribonuclease (RMRP),^[1,2]^ The clinical features of CHH include short-limbed dwarfism, as well as a range of extracutaneous manifestations such as hypoglycemia, gastrointestinal dysfunction, immunodeficiency, anemia, and increased risk of malignancy, among others^[3]^ The most common phenotype is characterized by disproportionate short limb dwarfism with short, thickened long bones, and it is usually found in newborns and occasionally in the prenatal stage^[1]^ The condition was initially identified by McKusick within the Old Order Amish community, a religious isolat,^[4]^ and it was subsequently found to be particularly prevalent in the Finnish population.^[5]^ CHH is extremely rare in the general population and there is no precise global incidence, but it has been calculated that Amish and Finnish carriers have a frequency of up to 1:19 and 1:76, respectively.^[6]^

The *RMRP* gene, which causes CHH, encodes a long-stranded noncoding RNA (lncRNA) and was the first nuclear noncoding RNA gene discovered to cause a genetic disease. Composed of ten protein subunits, it is a eukaryotic ribonuclease endonuclease necessary for the correct processing of ribosomal RNA, mitochondrial RNA, and certain messenger RNAs.^[7]^ Mutations in *RMRP* can lead to inherited developmental disorders such as CHH and dysplasia, but the mechanism is not fully understood. Here, we report a rare biallelic *RMRP* mutation causing CHH in a Chinese boy.

## 2. Case presentation

A 19-month-old boy was admitted to the outpatient clinic in July 2025, with persistent short stature since birth. The patient exhibited postnatal growth retardation and poor feeding habits. Previous evaluations showed normal calcium-phosphorus metabolism, thyroid function, parathyroid hormone, IGF-1, and hepatic/renal function. He was a first child, delivered by cesarean section at term (BW 3.3 kg, BL 50 cm). Maternal amniocentesis with the whole-exome sequencing (WES) at 24 weeks (due to fetal humeral/femoral shortening) revealed no pathogenic variants. No perinatal complications, surgical trauma, or seizures occurred. The boy sat independently at 6 months, walked at 13 months, and spoke “papa/mama” meaningfully. Both parents were in good health.

### 2.1. Physical examination

The patient exhibited severe short stature (height 70 cm, <−3 SD; weight 9.75 kg), with proportionate short limbs and sparse scalp hair. Neurological assessment revealed intact visual and auditory tracking, prompt response to his name, and preserved joint attention. No dysmorphic features were noted: absence of café-au-lait spots, jaundice, lymphadenopathy, blue sclerae, or high-arched palate; normal interpupillary distance and ear positioning, with no single palmar crease. Cardiopulmonary auscultation was unremarkable, and the abdomen was soft without tenderness. Musculoskeletal examination indicated normal limb muscle strength/tone, physiological reflexes present, and no pathological reflexes.

### 2.2. Laboratory and imaging results

Laboratory studies showed normal results for: complete blood count, urinalysis, stool analysis, hepatic/renal function, cardiac enzymes, electrolytes, thyroid/parathyroid function, IGF-1, and vitamin D levels.

**Figure 1.**
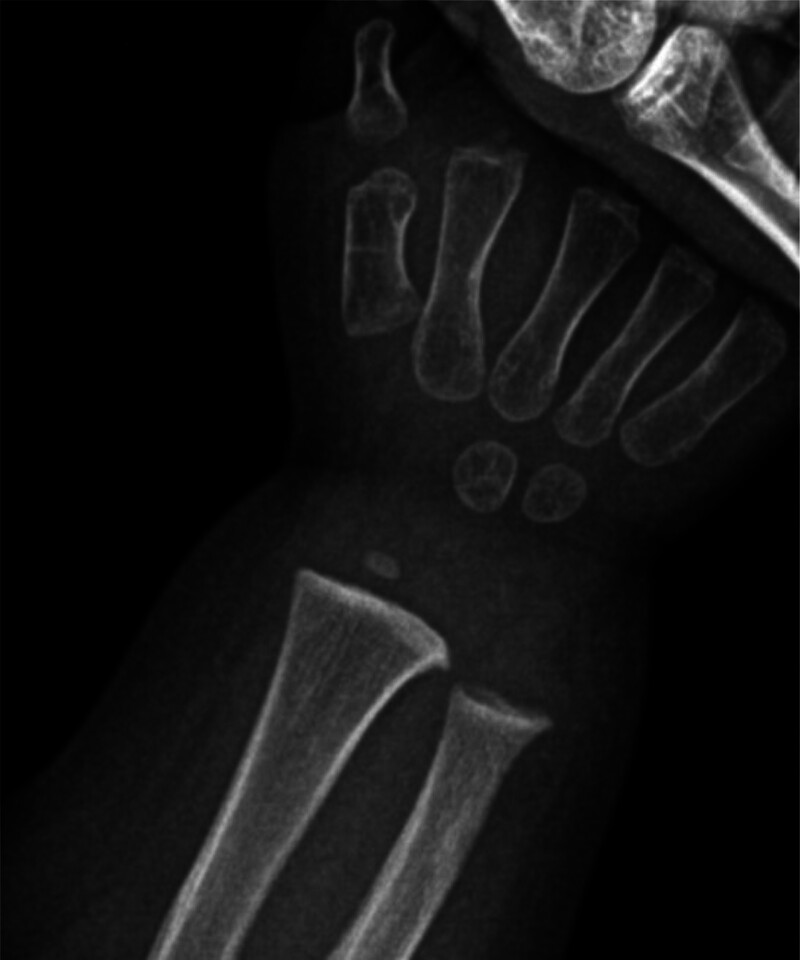
Left wrist X-ray: shortened metacarpals, metaphyseal flaring of distal radius/ulna, osseous remodeling. The left wrist joint and bone changes of the ulna and radius should be evaluated to exclude cartilage dysplasia.

**Figure 2.**
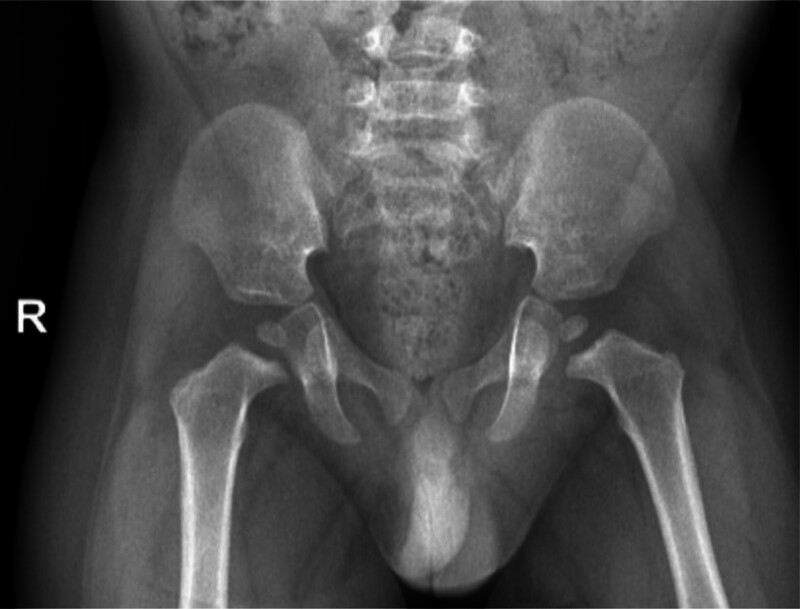
Hip X-ray: bilateral mild coxa vara, widened joint space, irregular femoral head contour with heterogeneous density. Changes in both hip joints warrant evaluation for exclusion of chondrodysplasia.

Radiographic findings: Left wrist radiograph revealed shortened metacarpals, metaphyseal flaring of the distal radius/ulna, osseous remodeling. Left wrist joint and bone changes of the ulna and radius were excluded from cartilage dysplasia (Fig. 1). Hip radiograph revealed bilateral mild coxa vara, widened joint space, irregular femoral head contour with heterogeneous density, and changes in both hip joints, pending exclusion of chondrodysplasia (Fig. 2).

### 2.3. Genetic analysis

After obtaining the consent of the child’s parents, we performed whole-exon gene sequencing for the child and parents on August 1, 2025. Family-based WES revealed compound heterozygous variants in the *RMRP* gene: NR_003051.3: n.-21_-9dup and n.5C > T. Classified as pathogenic and likely pathogenic, respectively, according to the ACMG guidelines, these variants confirmed the diagnosis of CHH.

Based on the *RMRP* mutation and clinical manifestations, the child was diagnosed with CHH.

## 3. Discussion

Mutations in RMRP cause CHH by disrupting the function of RNase MRP RNA, which affects multiple organ systems^[2]^ Research has demonstrated that mutations in the *RMRP* gene can disrupt the cell cycle, particularly by inducing delays during the transition from the G2 phase to mitosis. This arrest in the cellular cycle may result from aberrant downstream gene expression triggered by *RMRP* mutations, which subsequently impacts cell proliferation and differentiation.^[8,9]^ Additionally, mutations in the *RMRP* gene have been linked to immune system dysfunction, notably manifesting as significant impairments in T-cell activation. This phenomenon is likely attributable to disruptions in ribosome synthesis.^[10,11]^ In animal models, deletion or mutations in *RMRP* can induce phenotypes analogous to CHH. For example, in zebrafish models, *RMRP* mutations result in chondrodysplasia and abnormal ossification processes, which are associated with the upregulation of Wnt/β-catenin signaling pathways^[12]^ This dysregulation of signaling pathways may represent a key mechanism underlying the skeletal dysplasia caused by *RMRP* mutations. Furthermore, mutations in *RMRP* also impair chondrocyte hypertrophy and differentiation, thereby underscoring its critical role in skeletal development.^[13]^ So far, 133 mutations have been described in patients with CHH, of which 90 have been identified in RMRP transcripts and the others in RMRP promoters, with most mutations occurring in highly conserved regions.^[14]^

Our patient mainly presented with short stature and sparse hair. First, we reviewed the fetal development and birth history of the child. The father and mother of the child were married and conceived at an appropriate age. The fetal humerus/femur was shortened at 24 weeks of gestation, the parents underwent amniocentesis and WES, but no pathogenic gene variants were found. Moreover, other developmental and birth histories were normal. Second, we reviewed the growth and development milestones of the child. His nerve, language, and motor development histories were basically within the normal range, as were blood and biochemistry analyses, which ruled out endocrine and metabolic diseases such as congenital hypothyroidism and growth factor deficiency. At the same time, the child did not have developmental deformities, facial abnormalities, or other problems; therefore, genetic lesions such as Down syndrome and Noonan syndrome could be ruled out. However, when checking the bone age of the left wrist and hip joint, it was found that the child had long-term distal achondroplasia, which made us suspect rare genetic metabolic diseases again. After obtaining consent from the parents, we performed a whole-exon gene test on August 1, 2025. The results indicated that the child had a compound heterozygous variant of the *RMRP* gene, RMRP(NR_003051.3) n.-21_-9dup and n.5C > T. Both sites have been reported in the existing literature, and the ACMG guidelines suggest that they are pathogenic and likely pathogenic, respectively. Due to the elevated tumor predisposition associated with CHH, and because growth hormone therapy is currently contraindicated, no disease-specific interventions have been initiated. Children should be followed up every 3 to 6 months in a specialist clinic.

## 4. Conclusion

Regarding the case we reported, perhaps due to our lack of medical experience, although we have not been able to directly confirm the diagnosis based on the child’s symptoms, physical examination and routine auxiliary examination, but due to the particularity of the child’s symptoms, we have not ruled out the possibility of genetic and metabolic diseases, so we did a whole-exome genetic test for the child and his family in time to confirm the diagnosis. However, for this case and most genetic metabolic diseases, our biggest problem is that after the diagnosis is clear, we cannot give effective treatment plans for children and families, what we can do is to do long-term follow-up, pay attention to the research progress of such diseases, and we also hope that 1 day we can bring hope for cure to children and families through gene therapy and other methods.

Here, we reported a rare case of CHH caused by *RMRP* gene mutations. Although both mutations have been reported individually, their occurrence together in the same individual is very rare in existing case reports. In short, our case report added to the existing clinical phenotypic profile of this gene mutations, further highlighting that genetic metabolic diseases are relevant in the area of children’s growth and developmental disorders, warranting the attention of every clinician.

## Acknowledgments

Thanks to the children and their parents for their contribution to this study.

## Author contributions

**Formal analysis:** Yangfan Qi, Xiaoyu Sun.

**Investigation:** Kai Jiang.

**Resources:** Shuangzhu Lin

**Supervision:** Kai Jiang.

**Validation:** Wanqi Wang.

**Writing – original draft:** Qiandui Chen, Wanqi Wang, Xinyu Zhou.

**Writing – review & editing:** Qiandui Chen, Xiaoyu Sun.
